# Creating a service system architecture for people living with multiple long-term conditions

**DOI:** 10.1016/j.fhj.2026.100550

**Published:** 2026-07-07

**Authors:** Alison McKay, Francisco Tapia Lara, Sharanjit Boughan, Jason Broch, David Edeson, Max Henderson, Omar Huerta, Richard Irvine, Sarah R. Kingsbury, Lindsay McFarlane, Amy Rebane, Dan B. Trowsdale, Philip G. Conaghan

**Affiliations:** a School of Mechanical Engineering, University of Leeds, United Kingdom; b Leeds Institute of Rheumatic and Musculoskeletal Medicine, University of Leeds, and NIHR Leeds Biomedical Research Centre, United Kingdom; c NHS West Yorkshire Integrated Care Board, Leeds, United Kingdom; d Oakwood Lane Medical Practice, Leeds, United Kingdom; e Leeds Institute of Health Sciences, University of Leeds, United Kingdom; f Leeds City Council, Leeds, United Kingdom

**Keywords:** Patient journey, Multi-disciplinary teams (MDT), Remote monitoring, Service co-design, Patient involvement

## Abstract

**Purpose:**

To identify barriers and improvement opportunities for the effective management of care for people with multiple long-term conditions (MLTCs) and propose a service system architecture to ensure an integrated approach to achieving improved outcomes.

**Methods:**

A systems engineering process was used to derive stakeholder needs from multimorbidity case studies. An abductive analysis provided principles for system design. Hackathons using LEGO® SeriousPlay® helped co-create a service system architecture with patients and service providers.

**Results:**

Stakeholder needs captured through three metaphors (spaghetti, barriers, elephants in rooms) reflected concerns with current solutions; six principles for designing MLTC care services addressing service user and provider needs, mental health considerations, costs and system integration; and a service system architecture blueprint for MLTC care pathways.

**Conclusions:**

The novel system architecture increases equality and equity and provides a framework for holistic MLTC care spanning multiple clinical specialties and disciplines.

## Highlights

• MLTC service needs captured in metaphors: spaghetti, barriers, elephants in rooms.
• Design principles covering costs, benefits, mental health and system integration.
• Architecture for holistic care across multiple clinical specialties and disciplines.

## Introduction

The need for joined-up thinking in the care of people living with multiple long-term conditions (MLTCs) is widely recognised, with calls to redesign multi-condition healthcare services and shift their focus from single to multiple diseases.^1^ Further, such services need to integrate the care of physical and mental illnesses because people with MLTCs are more than twice as likely to experience mental health problems, and this risk is consistently associated with worse outcomes. Baird *et al*,^2^ among others, argue for a whole-systems approach to move beyond disease-specific care models. International examples including Staten Island^3^ and the Netherlands,^4^ along with the neighbourhood health model described in the NHS 10-year health plan,^5^ demonstrate the potential of holistic, community-based strategies for person-centred services.

By bringing together the needs of multiple stakeholders, systems engineering^6^ provides an inclusive and holistic approach to the design of complex systems such as MLTC care pathways. The aim of this study was to use systems engineering principles to identify barriers and improvement opportunities for the effective management of care for people with MLTCs and consequently propose a service system architecture to enable such care to be provided with equality and equity.

## Method

MLTC service systems are typical of the most complex kind of system^6^ in that they both impact and are impacted by multiple clinical disciplines and specialties, and economic, social and environmental factors. The design of new MLTC service systems can, therefore, be seen as a ʻwicked problem’^7^: no individual can fully understand the whole problem (Finitude); it is difficult to link actions to consequences because the problem and potential solutions are highly interconnected (Complexity); and the behaviour of the system is governed by human and organisational behaviours that influence the feasibility and effectiveness of solutions (Normativity). A consequence, especially in safety-critical systems such as health and social care, is that innovations are most likely to succeed if introduced incrementally. This provided the underlying rationale for the methods used.

**Fig. 1:**
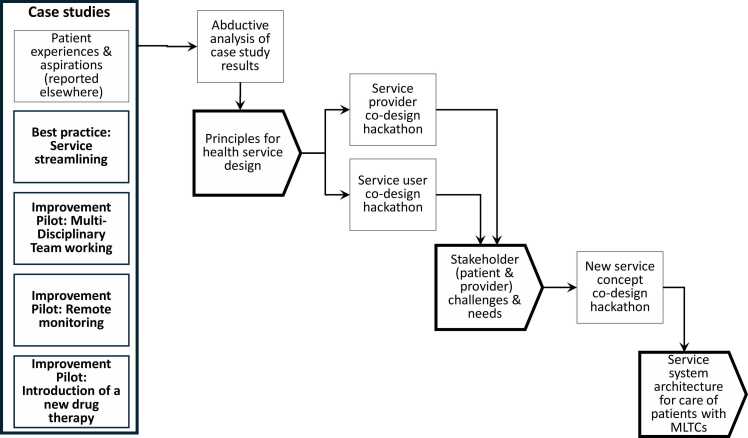
Study design and key outcomes (shown with bold outlining).

The Royal Academy of Engineering^6^ provides six principles for integrated system design (Table 1). For a wicked problem such as this, these principles formed the theoretical foundation for the research methods employed. The research centred on three of the most deprived communities in Leeds, which is demographically typical of many UK cities, with 21% of people living in relative poverty after housing costs.^8^ Our overall process (Fig. 1) used five case studies (four reported in section *Case studies* and the fifth on patient and public involvement, manuscript in development) to inform an abductive analysis that, coupled with experience of best practice,^9^ led to the identification of principles for the design of MLTC service systems. The case studies included an MLTC streamlining project on annual reviews that had been recently implemented in primary care, and three improvement pilots that used different solution principles (remote monitoring technology, a new drug therapy and multidisciplinary team working) to improve service offerings for patients with MLTCs including diabetes, kidney disease and heart failure. For each pilot, a review of literature and discussions with project leaders^10^ informed the design of a meeting where benefits and drawbacks of each solution principle were identified; conclusions from these meetings were documented and reviewed with participants and used to derive service design requirements (Table 2). Together these informed three hackathons where LEGO® SeriousPlay®^11^ was coupled with service blueprinting^12^ to enable co-creation of design requirements and a service system architecture. A summary of the participants in each pilot project meeting and hackathon is provided in the Supplementary Information. In the pilot project meetings, all participants contributed to a single discussion, whereas in the hackathons, the participants were split into groups designed to maximise the mix of expertise and organisations within each group.

**Table 1:** Principles for integrated system design and how addressed in this paper.

Principle from Royal Academy of Engineering	How addressed
Debate, define, revise and pursue the purpose	Learning from the case studies. Metaphors developed through the hackathons Hackathon models of current and ideal services PPIE activities.
Think holistic	Use of,^9^ which includes whole-system goals and aspirations, through the whole process ensured whole system design goals were considered throughout. The research team included people from multiple specialties and disciplines, including mental health specialists and health economists.
Follow a systematic procedure	We used a version of the systems engineering vee model,^6^ adapted for use in the design of non-physical products such as services. This was integrated into the research process presented in Fig. 1.
Be creative	The hackathon process ensured creativity from all participants.
Take account of the people	The hackathons included both service users and providers. PPIE was integrated through the entire activity.
Manage the project and the relationships	The case studies included people from different organisations and roles; our methods ensured effective communication at all stages. The hackathon process allowed people to be heard, so enhancing relationships within the project and with wider stakeholder communities.

**Table 2:** Design requirements.

Requirement	Primary case studies	Rationale
Services that can be configured to reflect each patient’s LTC cluster and disease processes.	Streamlining of annual reviews	Treating each patient holistically benefits both patients and service providers
Given an appropriate collection of services, there is a need for a coordination capability that is tailorable to the needs of individual patients and their disease processes.	Streamlining of annual reviews	Effective coordination of care makes services easier for patients to use.^13^, ^14^
Personalisation to reflect the needs and capabilities of individual patients and their disease processes.	Remote monitoring	For example, one patient’s normal reading for one metric was outside the range in the app, which led to unnecessary (for this patient) warnings that raised his anxiety levels and caused unnecessary interactions with services.
Integration across specialties.	Remote monitoring, new drug therapy	For example, the way in which the remote monitoring app was originally built meant it monitored individual conditions in isolation. Applying it in different single condition pathways would result in multiple monitoring schedules and so duplication and unnecessary complexity for patients.
Integration with existing IT systems.	Remote monitoring	For example, multiple apps need to link with GP and hospital systems.
Integration into clinical practice.	Remote monitoring, MDT	Escalation pathways need to be defined for individual patients.
Specification of an MDT needs to include details of the kind of patient data to be used, and the disciplines and clinical specialties to be included.	MDT	MDTs can take many forms and clarity is needed to ensure a given MDT has appropriate capabilities for its intended use.
Need for economic models. that include costs, benefits and adverse effects covering patients and the people and organisations involved in their care.	MDT, remote monitoring, new drug therapy	Current economic models cover financial costs and benefits for providers but not patients and other service users. Further, costs of adverse effects are typically excluded.
Need for service designs that make explicit both provider and patient pathways in order that they can be evaluated before implementation.	New drug therapy	For example, there was a need to evaluate service design options with respect to service provider workloads and accessibility for patients.

## Results

### Case studies

#### Streamlining of annual reviews

In usual care, reviews were carried out by the primary care practice for each of a patient’s LTCs annually based on diagnosis date; service providers would deliver multiple annual reviews to each patient, resulting in complexity, duplication and inefficiencies. Streamlining this service involved changing to a single annual MLTC review based on the patient’s date of birth. Service blueprints illustrating this are provided in the Supplementary Information. Although not quantified, staff reported anecdotal evidence of improvements in both patient and provider experiences that aligned with the blueprints.

#### Remote monitoring of patients with MLTCs including diabetes

This case built on a pilot project exploring the value to primary care teams of remote monitoring technologies^15^ in enabling patients to become more involved in self-management of their LTCs. The project used a commercially available app to allow patients with MLTCs including type 2 diabetes (recruited from two GP practices) to track their own health metrics (such as blood pressure and oxygen levels) at home and alert healthcare providers if anomalies were found. Reported benefits of remote monitoring technologies include improved disease knowledge for patients^16^ and promotion of day-to-day self-management.^17^ There are many suppliers of such technologies; however, their use in the NHS is typically driven from secondary care specialties and so focus on individual LTCs. Patient concerns included the emotional burden of constant tracking^18^ and hesitancy in using new technologies,^17^ in part driven by varied support in using new technologies which, in turn, resulted in increased staff workloads. Evidence supporting the effectiveness and scalability of such interventions remains limited.^19^ This case confirmed some of these issues, with patients reporting increased confidence and autonomy, although there was mixed data on healthcare utilisation with an increase for some patients.

#### Multidisciplinary team (MDT) working

MDT interventions have been associated with reduced hospital readmissions.^20^ This case built on learning from a recently completed pilot exploring the use of MDTs to reduce hospital readmission rates and improve quality of life for heart failure patients. For a period of 8 months, specialist clinicians, pharmacists and nurses from secondary care held virtual MDT meetings with primary care staff across six primary care networks in Leeds. The MDTs considered ‘paper patients’ (to avoid issues with sharing of personal data) who were identified and characterised by primary care providers. During the project, over 600 patients were reviewed, and MDT discussions led to therapy changes in over half and diagnostic tests for a third.

MDT working can take many forms, characterised across three dimensions: the patient data used (ranging from anonymised through pseudonymised to identified patients), the range of disciplines included (doctors, nurses, pharmacists, allied health professionals and carers), and the range of clinical specialties covered. This case study used pseudonymised patient data and each MDT included multiple professions with cardiac expertise. However, a key barrier to scale-up lay in the challenges of quantifying economic costs and benefits covering both patients and the people and organisations involved in their care that are necessary precursors to the development of cases for change.

#### Introduction of a new drug therapy

New drug therapies are introduced regularly based on evidence from clinical trials demonstrating their efficacy and cost-effectiveness. This case built on a pilot project that was co-funded by a pharmaceutical partner, ran for 12 months and targeted patients with chronic kidney disease (CKD) suitable for treatment with sodium glucose cotransporter 2 inhibitor (SGLT2i) drugs. SGLT2i treatments reduce the risk of CKD progression, major cardiovascular events and mortality, and the drugs are approved in the UK for the management of CKD in patients with type 2 diabetes.^21^ As patient benefits were unlikely to manifest themselves until several years later, the goal of the pilot was to optimise care for CKD patients by initiating SGLT2i treatment.

Within the pilot, service providers followed pre-defined protocols^22^, ^23^, ^24^ that identified key steps for service delivery. However, two important challenges were encountered.

1) Identifying patients with early-stage CKD: because CKD is often asymptomatic until late in its progression.
2) Implementation of available guidance: this was translated into flowcharts that allowed service providers to visualise key process steps and anticipate necessary changes in staff workloads.

This case highlighted factors outside the scope of this paper: the need for accurate coding of patients in health records, and alignment of project length with timescales of potential benefits. Within scope was a need for improved understanding of the impact of the introduction of the new drug on both service providers and users. For providers, the flowchart captured key steps from their perspective. However, the burden on patients, such as the need to attend appointments to provide samples to enable titration of drugs, was not visible.

### Principles for MLTC health service design

#### Include the target MLTC service users in the design process

We ensured that the lived experiences of people with physical-mental MLTCs from our target communities were integral to the research to ensure that their views were represented on how things look in practice and what they see as the barriers and enablers. We did this through community engagement and the primary care networks. By meeting people where they were and offering flexibility in how they could get involved (for example, small group meetings in known community locations, 1:1 informal chats and tailored information), we were able to offer inclusive opportunities for involvement. This also helped build trust with the research team, and we were able to deliver a hackathon with patients and carers in a way that was accessible for their needs to ensure that their input was included in our final outputs.

#### Consider the specific needs of target service users

We built on previous work with services users to enable participatory design.^25^ Four improvement opportunities for MLTC services had been identified^9^: provision of accessible and timely information to patients and carers; more effective communication (ideally, named workers); services geared towards the needs of people living in areas of higher deprivation where the prevalence and impact of MLTCs is higher; and ensuring that expectations with respect to self-management reflect socio-demographic and -economic factors, such as people living in more deprived areas being more likely to be exposed to smoking.

#### Consider patients’ mental health conditions

There is a bidirectional relationship between mental health and LTCs, which intensifies with the number and severity of LTCs. Patients with MLTCs and mental health conditions tend to have more, longer-lasting symptoms, reduced adherence to treatment plans, increased risk of hospital admission and longer hospital stays.^26^ There is therefore a need to include specialists on mental health in the context of physical ill health. In our case studies, four mental health factors were considered: prevalence in target group of service users and effects on recruitment (primarily in recruitment of participants to co-design activities) and, in the design of services, acceptability and efficacy for patients with mental health conditions. Overall, both greater and lower engagement with primary care impacts how patients are assessed by GPs and whether they attract diagnoses.

#### Include all relevant people involved in service provision in the design process

Aujla *et al*’s recent review highlighted the need for whole-systems approaches that include both health and social care.^27^ Our case studies included service providers from a range of organisations with both expertise and specialist knowledge of our target users, including community organisations where people have access to support from those with whom they have trusted relationships. A general challenge for the provision of services for MTLCs lies in ensuring inclusion of experts and specialists relevant to the LTC clusters of individual patients, meaning that service design solutions need to be tailorable to ensure this.

#### Capture all elements of cost related to care and care pathways

While it is important to capture both direct and indirect costs related to the delivery of care and care pathways,^28^ particular challenges are experienced by people from more deprived communities, where the time and costs of engaging with services becomes prohibitive. Economic assessments that capture both costs and benefits of proposed innovations for both users and providers are essential to scale-up and evaluation of service innovations.

#### System integration: consider mechanisms for current and future integration

The importance of integration of new solutions with existing ones was most apparent in the remote monitoring case study. From a service provider perspective, the app was designed around monitoring single LTCs, and a patient with MLTCs could end up with more than one app and multiple devices to record similar data for different LTCs. This, coupled with issues related to the integration of app readings with current healthcare IT systems, highlighted a need for broader aspects of integration, such as organisational and service integration, to be included in commissioning of third-party solutions such as remote monitoring technologies.

### Stakeholder challenges and needs

Two hackathons were held, one with service providers and the other with service users, to build understanding of the challenges they experience and their needs for future services. This resulted in metaphors and barriers to equity (Table 3) which were used as system-level design requirements.

**Table 3:** As-is and to-be service system scenarios.

Metaphors	Barriers to equity	Representing as-is service	Impact on patients (to-be service)	Impact on providers (to-be service)
Spaghetti	Complicated service pathways with varied communication methods	Complicated patient and carer pathways that rely heavily on referrals that, in turn, cause delays, additional work (eg related to managing appointments) leading to avoidable A&E visits	Simpler journeys for patients that take less time so freeing up time for work and other activities	Simpler journeys for staff that take less time so freeing up time for prevention work … paving the way for early identification and intervention^28^: Goal 2
Fences and walls	Access needs not being met	Barriers to engagement, eg, services that demand levels of mobility that patients do not have or cannot afford	Fewer barriers to patient engagement by designing pathways reflecting right person, care, place and time	Improved engagement by patients – leading to reduced preventable unplanned care use across all health setting^28^: Goal 1
Elephants in rooms	Atomistic approach taken meaning that whole patient and their carers not considered	Mental health regarded as separate from physical conditions despite interplays between them being well known	Changed mindsets: to mental health being an integral part of physical-mental multimorbidities	

### A service system architecture for patients with MLTCs

**Fig. 2:**
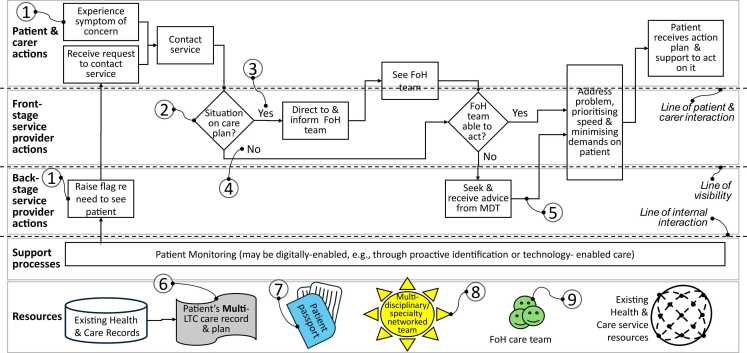
NOCAM-based service system architecture, (1) Triggers for patients to enter the service; (2) Check the patient’s MLTC care record/plan and determines next step; (3) Situation is on the plan: the patient is advised by the front-of-house (FoH) team; (4) Situation not on the plan: FoH team seeks advice from the MDST; (5) Advice is then acted on by the FoH team; (6) MTLC care record/plan; (7) Patient passport; (8) Multidisciplinary and specialty networked team (MDST); (9) FoH team (FoH).

The service system architecture introduced in Fig. 2 resulted from the third hackathon that, in line with the need for whole-systems approaches,^27^ used socio-technical systems methods to define the future (to-be) scenario in Table 3 from two perspectives: service users and providers. A service blueprint^12^ of the system architecture is provided in Fig. 2. The blueprint captures service processes in four swim lanes (patient and carer actions, onstage actions, backstage actions and support processes) and involves the four key service subsystems detailed in Table 4.

**Table 4:** Four key MLTC service subsystems.

Service subsystem	Purpose	Rationale for inclusion
Multidisciplinary and multispecialty networked team (MDST)	Provides specialist advice on the patient’s cluster of MLTCs when needed. In contrast to a traditional MDT, which is a fixed group that meets to discuss cases, a networked MDT is a flexible, relationship-based system that enables the right people, to come together at the right time without relying on formal meetings.	Simplifies patient journeys by reducing the need for referrals and, through the composition of the MDST, creates opportunities for more holistic management of mental and physical conditions which, in the longer term, has the potential to change mindsets of all involved.
Front-of-house team (FoH)	Provides a single point of contact for MLTC patients.	Simplifies patient journeys by incorporating care coordination as part of the service.
MTLC care record/plan	Building on the Richmond Group’s Collaborative Care and Support Planning approach,^1^ provides a holistic view of the patient to all people involved in service delivery.	The MLTC care record/plan of draws together data from patient records in different parts of the health & care system network to provide a holistic view for service providers without duplicating the data itself.
Patient passport	Provides data on patients’ personal circumstances, eg, work status, caring responsibilities, language and disabilities.	Enables personalisation of the service for patients. This, in turn, increases the likelihood of effective engagement with the service.

Triggers for patients to enter the service come from one of two sources: the patients themselves because they are experiencing symptoms that concern them or the service provider in response a flag, such as, for a routine review or raised in response to data from patient monitoring. In both cases, the patient passport ensures access needs are met through appropriate communication methods. When the service receives a contact from the patient, the front-of-house (FoH) team checks the patient’s MLTC care record/plan and determines next steps. If the situation is on the plan, then the patient is advised by the FoH team. Otherwise, the FoH team seeks advice from the MDST and the advice is acted on by the FoH team. In addition to the process structure, there are two non-process swim lanes: evidence and resources. The evidence lane highlights resources to be used in service evaluation and data that needs to be collected in service delivery; in Fig. 2 this lane is empty because the evidence to be collected will be implementation specific. The resources lane specifies the means to support service delivery and depends on available infrastructures. For example, Leeds has a city-wide shared care record that could underpin the MLTC care record/plan.

## Discussion

### Evaluation of service architecture

*Spaghetti*: The service blueprint makes processes visible and differentiates between patient and service provider actions. In addition, the line of patient and carer interaction highlights where users and providers need to interact with each other. Similarly, the line of internal interaction highlights where service providers need to interact with the delivery of support processes and systems. In this way, complicated patient and provider pathways become visible, so creating opportunities for improvement. For example, discussions highlighted the additional work and delays, for both users and providers, related to managing appointments associated with referrals to support services. The need for such referrals is reduced in our architecture by the MDST where service providers can seek advice related to the needs of individual patients.

*Fences and walls*: The differentiation between user and provider actions in the blueprint highlights the demands that a given service design places on both parties. For patients, especially those from the most deprived communities who have the highest frequency of unplanned activity, these can act as barriers to engagement. For example, services may demand levels of mobility that users do not have or resources (time and finances) that users cannot afford. Reducing spaghetti can address this to some extent but, beyond that, the FoH team and patient passport subsystems within the architecture provide opportunities for the removal of barriers to patient engagement by enabling the design of sustainable patient journeys which reflect the philosophy of right person, right care, right place, right time.

*Elephants in rooms*: Mental health is addressed through the composition of the MDST rather than the architecture itself. Specifically, the MLTC care record will include mental health as part of a patient’s disease cluster. Through this and the patient passport, both FoH teams and MDSTs will be aware of patients who have mental health conditions and how to best support them. While this can be seen as an improvement to services which currently focus primarily on physical LTCs, we recognise that mental health is still treated as a secondary illness rather than a comorbidity. For the longer term, mental health needs to be seen as a non-negotiable aspect of the philosophy underpinning the care of people with MLTCs. To this end, this new framework paves the way by facilitating changes in mindsets. Once mindsets have changed, further consideration of how mental health is best integrated into the service system architecture will be needed.

### Critique of methods used

The hackathon format was developed especially for this study and combined LEGO® Serious Play® with service blueprinting. This proved effective in both initial visualisation of service systems and provision of a platform to share potential solutions between multiple participants from diverse backgrounds. However, a limitation to this research was our viewpoint that the use (by both patients and providers) of MLTC pathways is a wicked problem and better suited to iterative improvements within the existing NHS system (first-order change) rather than sweeping (second-order) change. Although we did not attempt to limit participant suggestions in the hackathons, they and other aspects of the research were structured around the identification of improvement opportunities. As a result, by accepting the ambience of the current system, neither radical new system solutions nor limitations in what can be achieved through incremental change were considered.

The resources available, time and funding, meant that we did not evaluate the subsequent testing and implementation of the proposed architecture. However, we have participated in early experiments in three of the city’s most deprived communities, where anecdotal evidence suggests that the introduction of care coordinators and primary care-based MDSTs have improved the experiences of individual patients. Given appropriate resources, we would carry out an iterative cycle of service innovations coupled with developments of the four underpinning subsystems in Fig. 2. These cycles would include health economic evaluations to capture the full costs and benefits for both service providers and users.^29^ Alongside this, subsystem developments would use comprehensive health and social care data and emerging digital tools to underpin transformational step changes in modelling of both service utilisation and MLTC disease trajectories. In the longer term, these could form the basis of more radical, second-order changes that challenge current roles and structures, leading to the introduction of radically new service offerings.

## Conclusion

We have co-designed with MLTC patients and service providers a service system architecture that aligns with England’s NHS 10-year plan. Using a systems engineering approach allowed us to develop integrated multidisciplinary approaches shaped around a patient’s MLTCs to deliver current care capabilities holistically, rather than as parts of separate pathways. Through integrated MDTs, we demonstrated how aligned care capabilities (such as blood pressure management, lipid management and diabetes control) might be delivered. The approach allowed us to personalise MLTC management through better engineered treatment capability clusters, rather than aligning them to diagnosis-based pathways or services. Systems engineering can deliver a meaningful approach to service redesign and integration, either incrementally or disruptively for alignment of remote monitoring around clinical need rather than individual diagnosis pathways.

## CRediT authorship contribution statement

**Amy Rebane:** Writing – review & editing, Methodology, Investigation. **Dan B. Trowsdale:** Writing – review & editing, Methodology, Investigation. **Alison McKay:** Writing – review & editing, Writing – original draft, Supervision, Methodology, Conceptualization. **Philip G. Conaghan:** Writing – review & editing, Writing – original draft, Supervision, Methodology, Conceptualization. **Francisco Tapia Lara:** Writing – review & editing, Writing – original draft, Investigation. **Sharanjit Boughan:** Writing – review & editing, Methodology, Investigation. **Jason Broch:** Writing – review & editing, Resources. **David Edeson:** Writing – review & editing, Resources, Conceptualization. **Max Henderson:** Writing – review & editing, Resources. **Omar Huerta:** Writing – review & editing, Methodology, Investigation. **Lindsay McFarlane:** Writing – review & editing, Methodology, Conceptualization. **Richard Irvine:** Writing – review & editing, Resources. **Sarah R. Kingsbury:** Writing – review & editing, Methodology, Investigation.

## Ethics approval and consent to participate

This study was approved by the University of Leeds Research Ethics Committee under reference number MREC 23-065.

## Funding

This research was funded by the UK National Institute for Health Research and the Engineering and Physical Sciences Research Council through grant reference NIHR158156 (Using Population-based Systems to deliver improved and equitable health and care services for people with multiple long-term conditions: The Leeds SEISMIC HUB).

## Declaration of Competing Interest

The author(s) declared no potential conflicts of interest with respect to the research, authorship and/or publication of this article.

## Data Availability

Data resulting from the case studies will be available from the first author at *a.mckay@leeds.ac.uk* following a 24-month embargo to enable publication of a further related paper.
