# Supplementary material for: Creating a service system architecture for people living with multiple long-term conditions
Source: Future Healthc J. 2026 Jul 7;13(3):100550. doi: 10.1016/j.fhj.2026.100550 (PMC13449361; doi:10.1016/j.fhj.2026.100550)
Supplement: Supplementary file 1 [file mmc1.docx]

Appendix A: Supplementary information
A.1: Summary of the participants in each pilot project meeting and hackathon

A summary of the participants in each mapping meeting and hackathon is provided in Table 1. In the case study review meetings, all participants participated in a single discussion whereas in the hackathons the participants were split into groups designed to maximise the mix expertise and organisations within each group.

Table 1: Case study review meeting and hackathon participants

| Participant profession | Remote monitoring | MDT | New drug therapy | Service provider hackathon ^a^ | Patient & carer hackathon ^b^ | Research development hackathon ^c^ |
| --- | --- | --- | --- | --- | --- | --- |
| Primary care worker | 3 | 2 | 2 | 4 | 0 | 0 |
| Community care worker | 0 | 1 | 2 | 5 | 0 | 1 |
| Secondary care worker | 0 | 3 | 1 | 6 | 0 | 0 |
| Clinical academic | 1 | 2 | 2 | 0 | 0 | 2 |
| Commissioner | 4 | 4 | 4 | 4 | 0 | 2 |
| Patient & carer representative | 2 | 1 | 1 | 3 | 0 | 3 |
| Patient & carer | 0 | 0 | 0 | 0 | 7 | 0 |
| City Council | 3 | 1 | 0 | 2 | 0 | 0 |
| Systems engineering researcher | 4 | 5 | 4 | 0 | 0 | 2 |
| Healthcare researcher | 1 | 2 | 1 | 0 | 0 | 0 |
| TOTAL | 18 | 20 | 16 | 24 | 7 | 10 |
| ^a^ plus 15 observing and/or facilitating; ^b^ 5 patients & 2 carers (although both carers had MLTCs) plus 2 observing and 4 facilitating; ^c^ plus 5 observing and/or facilitating | | | | | | |

A.2: Service blueprints illustrating improvements through service streamlining

Information from the GP’s web site communicating a change in the management of annual MLTC reviews to patients, informed the development of the hypothetical service blueprint for a blood testing service in Figure 1.

Figure 1: Service blueprint for a simplified blood test as part of a single-LTC annual review

In the original service, annual reviews were carried out for each of a patient’s LTCs annually from the diagnosis date of the LTC. Implications of using such a single LTC-based solution for patients with three MLTCs is illustrated in Figure 2 where, for clarity, each LTC review is initiated within a 12-week window of the others. The blueprint highlights how complicated such combinations of services become for both patients and service providers. Inefficiencies, e.g., additional costs and time for both patients and providers, of using a single LTC-based model for patients with MLTCs are visible. The service improvement involved changing to an annual MLTC review based on the patient’s date of birth and is illustrated in Figure 3 where a significantly less complicated service structure can be seen.

Figure 2: Service blueprint schematic illustrating the impact of using the single-LTC review blood testing service for a patient with three MLTCs. NOTE: Each colour represents service process steps for a different LTC.

Figure 3: Service blueprint schematic illustrating the impact of using the MLTC review blood testing service for the same patient as in Figure 2. NOTE: Each step meets the requirements for all three MLTCs
